# Improvement of fertility parameters with Tribulus Terrestris and Anacyclus Pyrethrum treatment in male rats

**DOI:** 10.1590/S1677-5538.IBJU.2018.0843

**Published:** 2019-01-29

**Authors:** Dariush Haghmorad, Mohammad Bagher Mahmoudi, Pardis Haghighi, Paria Alidadiani, Ensieh Shahvazian, Parsova Tavasolian, Mahmoud Hosseini, Mahmoud Mahmoudi

**Affiliations:** 1 Laboratory Medicine School of Medicine Semnan University of Medical Sciences Semnan Iran Department of Pathology and Laboratory Medicine, School of Medicine, Semnan University of Medical Sciences, Semnan, Iran;; 2 Department of Immunology School of Medicine Semnan University of Medical Sciences Semnan Iran Department of Immunology, School of Medicine, Semnan University of Medical Sciences, Semnan, Iran;; 3 Department of Genetics Shahid Sadoughi University of Medical Sciences Yazd Iran Department of Genetics, Shahid Sadoughi University of Medical Sciences, Yazd, Iran;; 4 BuAli Research Institute School of Medicine Mashhad University of Medical Sciences Iran Immunology Research Center, BuAli Research Institute, Department of Immunology and Allergy, School of Medicine, Mashhad University of Medical Sciences; Iran; 5 Neuroscience Research Center School of Medicine Mashhad University of Medical Sciences Iran Neuroscience Research Center, Department of Physiology, School of Medicine, Mashhad University of Medical Sciences Iran

**Keywords:** Tribulus, Testosterone, Receptors, FSH, Receptors, LH

## Abstract

**Objective:**

Anacyclus Pyrethrum (AP) and Tribulus Terrestris (TT) have been reported as male infertility treatment in several studies; however, in Iranian traditional medicine these two plants are prescribed simultaneously. In this study, we aimed to determine the effects of AP and TT extracts both separately and simultaneously on the male Wistar rat fertility parameters.

**Materials and Methods:**

32 male Wistar rats were divided into 4 groups: Control, TT, AP, and AT treated groups. Treatment continued for 25 days and rats were weighed daily. Their testes were dissected for histological studies. Sperm analysis including sperm count, viability and motility were performed. Serum was obtained to evaluate testosterone, LH and FSH levels. Histological studies were conducted to study Leydig, and Sertoli cells, spermatogonia and spermatid cell numbers, and to measure seminiferous diameter and epithelium thickness.

**Results:**

Sperm count increased in all the treatment groups. Sperm viability and motility in AT and AP groups were elevated. TT and AT groups showed significantly increased testosterone level compared to control group (P=004, P=0.000, respectively) and TT, AP and AT treatment groups showed increased LH level (P=0.002, P=0.03 and P=0.000, respectively) compared to control, while only AT group showed increased FSH (p=0.006) compared to control. Histological studies showed significant increase of spermatogonia, Leydig and Sertoli cell numbers and epithelial thickness in AT group compared to other groups. All the treatment groups had higher number of Leydig, spermatogonia and spermatid cells.

**Conclusion:**

TT and AP improved sexual parameters; however, their simultaneous administration had higher improving effects on studied parameters.

## INTRODUCTION

Infertility refers to inability to achieve pregnancy after twelve months of regular and unprotected intercourse (1, 2). About 40-50% of infertilities are due to male sexual dysfunction, as one out of twenty men suffer from this issue, worldwide (3-6). The majority of infertile and sub fertile men have deficiency in the semen quality which is determined by low sperm numbers, sperm morphology and insufficient sperm motility. Other cases may appear by hormonal imbalances, anatomical problems and genetic defects (1, 2). Male fertility is the direct consequence of spermatogenesis, a multistep process in seminiferous tubules of testis, which is highly regulated by sophisticated hormonal signaling pathways (6, 7). Testosterone is the major androgen in the process of spermatogenesis promoting the maintenance of blood-testis barrier, Sertoli-spermatid adhesion and mature sperm release (8). Gonadotropin releasing hormone (GnRH) has a central role in controlling spermatogenesis. It performs its role by inducing follicle-stimulating hormone (FSH) and luteinizing hormone (LH) secretion from the anterior pituitary gland. LH stimulates adult Leydig cells to generate testosterone (7, 9), while FSH supports spermatogenesis by increasing Sertoli cell numbers and preventing apoptosis of spermatogonia and spermatocytes (9, 10).

Medicinal plants, as a traditional treatment, play a great role in remedies, due to their accessibility, availability and affordability, particularly in non-industrialized countries (11). Despite major advances in assisted reproductive techniques (ART), according to World Health Organization (WHO) estimation, almost 80% of world population trust on traditional health care (12). Plants empirical studies have shown their significant role in alternative therapy of sexual dysfunction (13).

Anacyclus Pyrethrum (AP) root, commonly known as pillitory, belongs to Asteraceae family. It is abundant in India and also found in Africa and Asia. AP has been known as male sexual stimulant. Ebn-e-Sina (14), great medieval Persian physician, prescribed it in Erectile Dysfunctions (ED) treatment. Other records indicate this herb constitute a major part of Polyherbal Ayurvedic Medicine (PAM), widely used for treating **male sexual dysfunction** (MSD) in the Indian subcontinent (15). In studies conducted by Sharma et al. on the effect of AP in rat sexual parameters, several observations were found, including enhanced sperm number, bodyweight, testis weight, seminal vesicle fructose and sexual hormones concentrations (16, 17).

Tribulus terrestris (TT) (Zygophyllaceae) is a Mediterranean plant, traditionally used against various ailments such as sexual inability, edemas, abdominal distention and cardiovascular diseases in India, China, Bulgaria and South Africa (18). A great number of studies have been performed on the effect of TT extract on sperm parameters in both human and animal models, leading to controversial results (13, 19-24).

There are several reports indicating TT effect on increasing body and sexual organs weight (13, 25). In a similar trend, Testosterone and LH elevation were observed (26). On the contrary, other studies reported no increase in testosterone and LH levels (21, 27). In 2014 Santos CA et al. observed that Tribulus terrestris was not more effective than placebo in improving symptoms of erectile dysfunction or serum total testosterone (19).

In several regions of Iran, including northern Khorasan, TT and AP are prescribed simultaneously as a traditional treatment for male sexual dysfunctions. Accordingly, this paper aims to evaluate serum levels of sexual hormones, sperm analysis and histological studies in male rats treated by separate extracts of Tribulus terrestris and Anacyclus Pyrethrum as well as mixture of both extracts as prescribed in traditional medicine.

## MATERIALS AND METHODS

### Plant material

Dried root of AP and flowers of TT were purchased from local market in Bojnourd (North Khorasan province), and it was recognized and authenticated in Botanical Systematic Laboratory, Department of Biology, faculty of science in Ferdowsi University of Mashhad.

### Preparation of extracts

The root and flowers were crushed to powder and were macerated in ethanol (70% v/v) for 72h at room temperature to prepare ethanolic extract. The yield for TT was 6.8% and for AP 7.2%. The salve extract was removed under reduced pressure until the volume reached to 50mL, then it was left in room temperature in petri dish and the dried mass was stored at 4^o^C.

### Animals

Thirty-two Healthy adult male Wistar rats (weight average 255±5g) were obtained from the Animal House of Mashhad University of Medical Sciences. They were kept in well-ventilated house conditions (temperature 28-31ºC and humidity 50-55%); photoperiod: 12h natural light and 12h dark; with free access to rat chow and tap water. This project was approved by the ethics committee of Mashhad University of Medical Science on the use of animal’s laboratory.

### Treatment

All rats were completely randomized into 4 groups containing 8 rats in each group. The groups were force fed as follows: 1) Control group was given Phosphate Buffer Saline (PBS) 2) TT Group was treated by 10mg/kg of Tribulus terrestris extract 3) AP group was treated by 100mg/kg of Anacyclus pyrethrum extract 4) AT group was administered by 10mg/kg of Tribulus terrestris extract and 100mg/kg of Anacyclus pyrethrum extract, simultaneously. The daily oral administration was carried out by the use of metal oropharyngeal cannula for 25 days. Each rat was administered by as much as 0.5mL of the solution. The extracts were dissolved in PBS; therefore, the control group was administered by the same volume of PBS as treatment group. The dose of administration for AP was chosen from Sharma’s study 100mg/kg of which had the best results and for TT 10mg/kg was chosen from Gauthaman study (17, 26).

### Body and organ weight

The body weight of animals was recorded daily. After 25 days, the animals were sacrificed; their testis and prostate glands were carefully removed and weighed.

### Sperm quality analysis

The cauda epididymis was directly isolated after cervical dislocation of the animals and placed in a Petri dish containing 1mL DMEM with 1% BSA. 1mL of the medium was placed in another petri dish, and a section of the cauda epididymis was isolated in this dish that remained in an incubator at 37°C to allow the spermatozoa to ‘swim out’ into the medium for approximately 10 second.

Sperm quality was analyzed by three parameters: sperm concentration, motility and viability. Sperm concentration was analyzed using hemocytometer method (15). One drop of cauda epididymal spermatozoa was diluted 1: 100 in DMEM supplemented with 1% BSA and placed in the center of the lower disc and further examined with a microscope. The diluted solution was put into the counting chamber and the sperm number was counted using hemocytometer under light microscope. Sperm motility was calculated by calculating the percentages of total and progressive motile spermatozoa using invert microscope and expressed as percentage of motility. Sperm viability was analyzed by Eosin-Nigrosin method under light microscope, where unstained spermatozoa counted as viable and stained spermatozoa counted as non-viable. The viability of spermatozoa was announced in percentage terms.

### Serum hormone analysis

Representative animals were bled by cardiac puncture, and the blood was allowed to clot at 4°C overnight. The samples were centrifuged, and the sera were collected and stored at-80°C until hormone analysis was performed.

The sera obtained from all groups were analyzed for testosterone, LH and FSH by ELISA using commercial kits. Testosterone was measured using Free Testosterone ELISA kit from IBL Germany. LH and FSH level were analyzed using Rat LH and FSH Assay kits from BioVendor, Czech Republic. Absorbance was read at 450nm using microplate ELISA reader (Stat Fax 2100, Awarness, and Phoenix, Arizona, USA). The concentrations of hormones were estimated from a standard curve generated with each run.

### Histology

After fixing the left testis in Bouin’s fixative, the testis were dehydrated using a gradient of ethanol and then cleared in xylene. It was then embedded in paraffin and microtomed into 5μm sections and stained using Hematoxylin and Eosin. Epithelium thickness, seminiferous tubule’s diameter, number of Leydig and Sertoli cells, number of spermatids and spermatogonia were assessed under a light microscope. Seminiferous tubule diameter and epithelium thickness were measured using ocular micrometer, measuring respectively 40 and 20 random seminiferous tubule diameters and thickness of their epithelium in each slide; afterwards the mean was calculated for each rat testis, the results are expressed as mean±SD for each group. Sertoli cells, spermatids and spermatogonia were counted in all seminiferous tubules of about 20μm in diameter, and then the mean was calculated for each rat; the results are expressed as mean±SD for each group. In order to calculate the Leydig cell number we counted 6 random areas of all slides, and then the mean was calculated for each rat. The results are expressed as mean±SD.

### Statistical analysis

One-way ANOVA was used for statistical comparison of the results. Fischer`s LSD multiple comparison test was applied to see if the differences were statistically significant. Significance was defined at the 0.05 level. SPSS 19 were used to analyze the data. Results are expressed as the mean±SD.

## RESULTS

### Body and sexual organ weights

**Figure 1:**
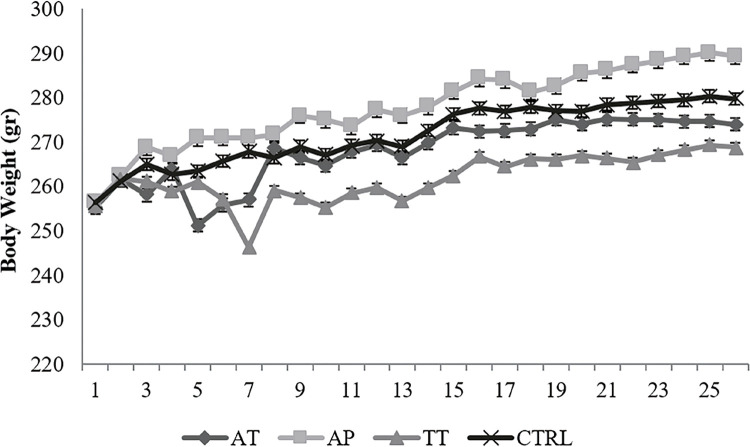
The body weight of animals was recorded daily.

An increase was observed in body weight of AP group, while a decrease was obvious in TT group compared to control group, however, AT group showed nearly the same weights as control group; however, none of the alterations were significant (Figure-1). Prostate weight showed elevation in all treatment groups but it was not statistically significant. Testis weight showed no significant change (Table-1).

An increase was observed in body weight of AP group, while a decrease was obvious in TT group compared to control group, however, AT group showed nearly the same weights as control group; however, none of the alterations were significant.

**Ctrl=**control group, **TT=***Tribulus terrestris*, **AP=***Anacyclus pyrethrum*, **AT=***Anacyclus pyrethrum and Tribulus terrestris*

**Table 1:** Sexual organ weights and sperm parameters taken in this study (mean ± SD, n=8)

Parameters	Mean ± SD	Range	P Value
**Sperm Count (No. of Sperm ×10**^**6**^ **/MM)**			
Control Group (n=8)	1.078 ± 0.621	0.457-1.699	
TT Group (n=8)	1.7225 ±0.482*	1.7225-2.2045	0.01
AP Group (n=8)	1.671 ± 0.404*	1.267-2.075	0.02
AT Group (n=8)	1.7245 ± 0.334*	1.3905-2.0585	0.01
**Prostate Weight /mg**			
Control Group (n=8)	63.7 ± 0.005	63.695-63.705	
TT Group (n=8)	75.3 ± 0.005	75.295-75.305	0.10
AP Group (n=8)	74.9 ± 0.005	74.895-74.905	0.11
AT Group (n=8)	70.9 ± 0.002	70.898-70.902	0.30
**Testis Weight /g**			
Control Group (n=8)	1.3412 ± 0.047	1.2942-1.3882	
TT Group (n=8)	1.275 ± 0.070	1.205-1.345	0.43
AP Group (n=8)	1.3588 ± 0.053	1.3058-1.4118	0.83
AT Group (n=8)	1.345 ± 0.060	1.2912-1.4012	0.96
**Prostate / Body (×10**^**3**^**)**			
Control Group (n=8)	0.2335 ± 0.054	0.1795-0.2875	
TT Group (n=8)	0.2797 ± 0.046	0.2337-0.3257	0.07
AP Group (n=8)	0.2605 ± 0.057	0.2035-0.3175	0.29
AT Group (n=8)	0.2535 ± 0.043	0.2105-0.2965	0.43
**Motility (%)**			
Control Group (n=8)	70.375 ± 2.800	67.575-73.175	
TT Group (n=8)	72.75 ± 2.829	69.921-75.579	0.59
AP Group (n=8)	90 ± 2.619*	87.381-92.619	0.00
AT Group (n=8)	82.75 ± 3.845*	78.905-86.595	0.01
**Viability (%)**			
Control Group (n=8)	83.875 ± 6.0460	77.829-89.921	
TT Group (n=8)	86.5 ± 6.563	79.937-93.063	0.09
AP Group (n=8)	87 ± 10.797*	76.203-97.797	0.05
AT Group (n=8)	90.75 ± 10.403*	80.347-101.153	0.00

ANOVA followed by Fischer`s LSD multiple comparison test. Data are presented as mean ± SD

* Significant results

**Ctrl=** control group; **TT=***Tribulus terrestris*; **AP=***Anacyclus pyrethrum*; **AT=***Anacyclus pyrethrum and Tribulus terrestris*

### Sperm parameters

Sperm count showed a significant increase (55-57%, increase) in all treatment groups compared to control group; however, none of the treatment groups showed significant change compared to each other. AT and AP treated groups showed significant elevation in their sperm viability (P=0.000 and P=0.05, respectively), however the increase in AT treated group`s viability was significant compared to AP and TT treated groups (P=0.03 and P=0.01, respectively). Motility had significant increase in AT and AP treated groups (Table-1).

### Hormonal levels

**Figure 2:**
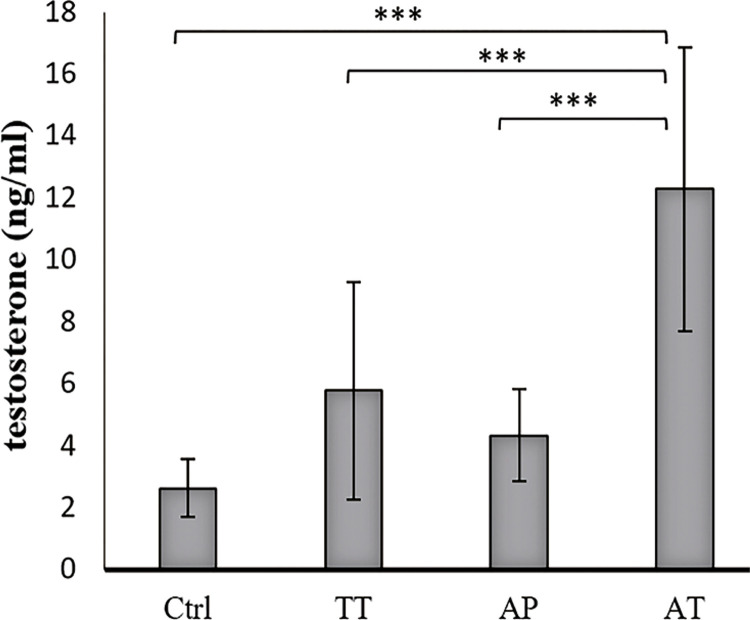
Effect of AP, TT and AT extracts on serum level of testosterone (mean ± SD).

**Figure 3:**
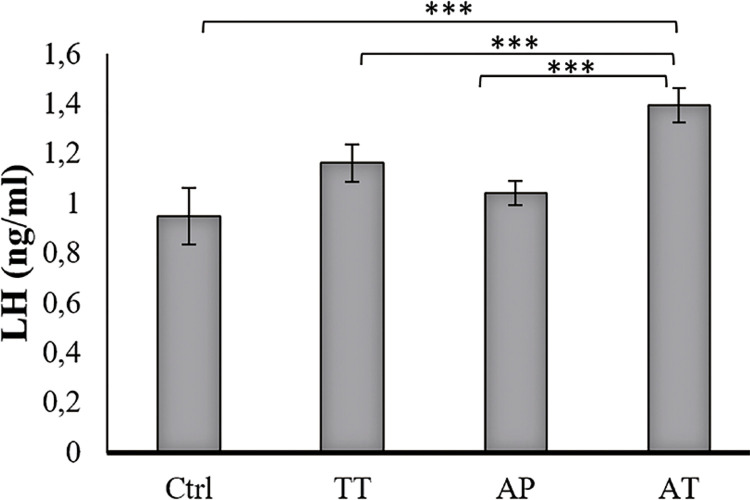
Effect of AP, TT and AT extract on serum level of LH (mean ± SD).

**Figure 4:**
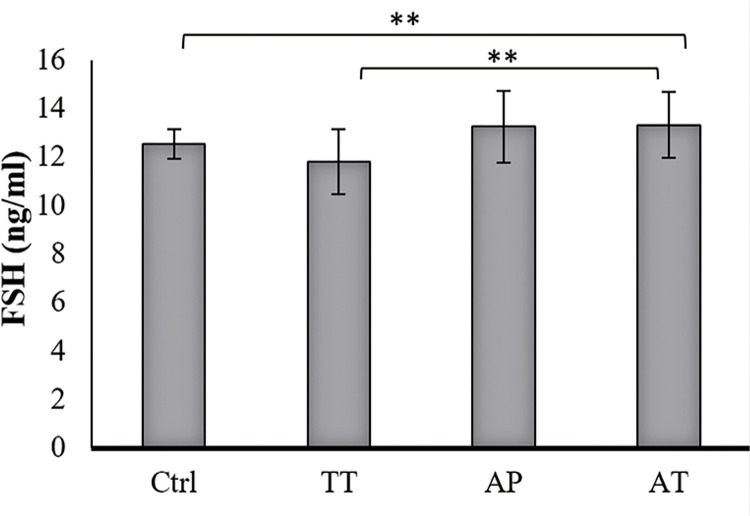
Effect of AP, TT and AT extract on serum level of FSH (mean ± SD).

Testosterone level was increased significantly in AT treated group compared to AP, TT and control groups (P=0.000, P=0.000 and P=0.000, respectively). TT group showed significantly increased testosterone level compared to control group (P=0.004). Increased testosterone level was observed in AP treated group as well, but it was not significant (Figure-2). LH levels increased significantly in AT treated group compared to AP, TT and control groups (P=0.000, P=0.000 and P0.000, respectively); it was also significant, in lower scales, in TT and AP groups in comparison to control group (P=0.002 and P=0.03 respectively) (Figure-3). FSH level was significant only in AT group compared to control group and TT group (P=0.000, P=0.000, respectively) (Figure-4).

The sera obtained from all groups were analyzed for testosterone, by ELISA using Free Testosterone ELISA kit from IBL Germany. Testosterone level was increased significantly in AT treated group compared to AP, TT and control groups (P=0.000, P=0.000 and P=0.000, respectively). TT group showed significantly increased testosterone level compared to control group (P=0.004). Increased testosterone level was observed in AP treated group as well, but it was not significant.

**Ctrl=**control group, **TT=***Tribulus terrestris*, **AP=***Anacyclus pyrethrum*, **AT=***Anacyclus pyrethrum and Tribulus terrestris.* *P <0.05, ** P < 0.01, ***p<0.001

The sera obtained from all groups were analyzed for LH by ELISA using Rat LH Assay kit from BioVendor, Czech Republic. LH levels increased significantly in AT treated group compared to AP, TT and control groups (P=0.000, P=0.000 and P0.000, respectively); it was also significant, in lower scales, in TT and AP groups in comparison to control group (P=0.002 and P=0.03 respectively).

**Ctrl=**control group, **TT=***Tribulus terrestris*, **AP=***Anacyclus pyrethrum***, AT=***Anacyclus pyrethrum and Tribulus terrestris***.** *P <0.05, ** P < 0.01, ***p<0.001

The sera obtained from all groups were analyzed for FSH by ELISA using Rat FSH Assay kit from BioVendor, Czech Republic. FSH level was significant only in AT group compared to control group and TT group (P=0.000, P=0.000, respectively).

**Ctrl=**control group, **TT=***Tribulus terrestris*, **AP=***Anacyclus pyrethrum***, AT=***Anacyclus pyrethrum and Tribulus terrestris***.** *P <0.05, ** P < 0.01, ***p<0.001

### Histology

**Figure 5:**
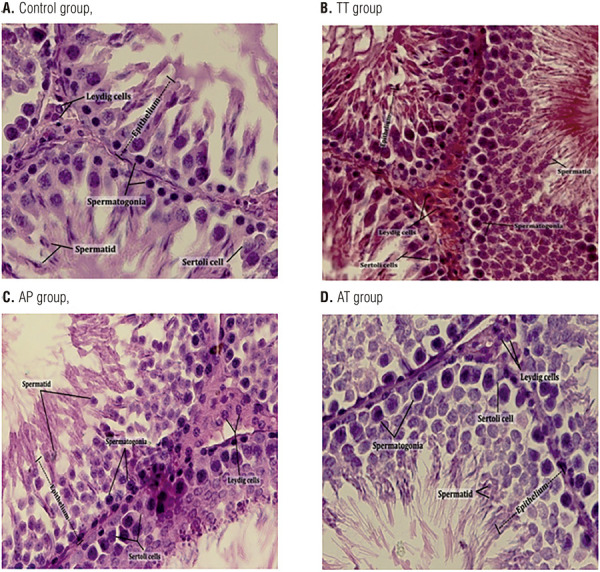
Comparative H&E staining of seminiferous epithelium demonstrated increased thickness and Leydig, Sertoli, spermatid and spermatogonia cell numbers in AT group.

Seminiferous epithelium thickness significantly increased in AT group compared to control group (P=0.04). Leydig, Sertoli, spermatid and spermatogonia cell numbers elevated significantly in AT group compared to all other groups. Seminiferous diameter did not show any significant changes among all groups (Figure 5 and Table 2).

H&E staining of seminiferous epithelium from all groups was performed. Left testis from each group, collected on day 25, fixed in Bouin’s fixative and embedded in paraffin. Five µm sections from different regions of testis from each of the groups were stained with H&E. Epithelium thickness, seminiferous tubule’s diameter, number of Leydig and Sertoli cells, number of spermatids and spermatogonia were assessed under a light microscope. Seminiferous epithelium thickness significantly increased in AT group compared to control group. Leydig, Sertoli, spermatid and spermatogonia cell numbers elevated significantly in AT group compared to all other groups. Seminiferous diameter did not show any significant changes among all groups (Additional data have been shown in Table 2).

**Table 2:** Seminiferous diameter, epithelium thickness and Sertoli, Leydig, spermatid, spermatogonia cell numbers taken in this study (mean ± SD)

Parameters	Mean ± SD	Range	P Value
**Seminiferous diameter (µm)**			
Control Group (n=8)	24.27 ± 0.61	23.66-24.88	
TT Group (n=8)	24.35 ± 1.50	22.85-25.85	0.89
AP Group (n=8)	23.10 ± 0.94	22.16-24.04	0.06
AT Group (n=8)	23.00 ± 1.58*	21.42-24.58	0.04
**Epithelium thickness (µm)**			
Control Group (n=8)	0.67 ± 0.04	0.63-0.71	
TT Group (n=8)	0.73 ± 0.9	0.17-1.63	0.13
AP Group (n=8)	0.66 ± 0.3	0.36-0.96	0.67
AT Group (n=8)	0.75 ± 0.10*	0.65-0.85	0.04
**Sertoli cell number**			
Control Group (n=8)	19.65 ± 2.12	17.53-21.77	
TT Group (n=8)	20.63 ± 2.39	18.24-23.02	0.36
AP Group (n=8)	19.50 ± 2.20	17.3-21.7	0.87
AT Group (n=8)	33.88 ± 4.39*	29.49-38.27	0.00
**Leydig cell number**			
Control Group (n=8)	12.00 ± 0.53	11.47-12.53	
TT Group (n=8)	14.25 ± 1.49*	12.76-15.74	0.00
AP Group (n=8)	13.88 ± 0.83*	13.05-14.71	0.01
AT Group (n=8)	18.75 ± 1.98*	16.77-20.73	0.00
**Spermatid cell number**			
Control Group (n=8)	52.13 ± 7.49	44.64-59.62	
TT Group (n=8)	56.87 ± 6.51	50.36-63.38	0.30
AP Group (n=8)	62.50 ± 5.35*	57.15-67.85	0.03
AT Group (n=8)	70 ± 13.89*	56.11-83.89	0.00
**Spermatogonia cell number**			
Control Group (n=8)	113.00 ± 19.49	93.51-132.49	
TT Group (n=8)	134.50 ± 14.15*	120.35-148.65	0.01
AP Group (n=8)	135.87 ± 3.74*	132.13-139.61	0.01
AT Group (n=8)	220.00 ± 17.42*	202.58-237.42	0.00

ANOVA followed by Fischer`s LSD multiple comparison test. Data are presented as mean ± SD

* Significant results

**Ctrl=**control group, **TT=***Tribulus terrestris*, **AP=***Anacyclus pyrethrum*, **AT=***Anacyclus pyrethrum and Tribulus terrestris*.

## DISCUSSION

The present study demonstrates effects of TT and AP either separately or together, on reproductive parameters of male Wistar rats. Measured sperm parameters, sperm count, mobility and viability, in AT and AP treatment group compared to controls were significantly influenced. Also, TT treatment alone showed effects on sperm count, which is consistent with previous studies (18, 28). Cell numbers and properties related to sexual Wistar rat organs were analyzed. Seminiferous diameter, epithelium thickness, Sertoli, Leydig, spermatid and spermatogonia cell number were significantly increased in AT group compared to controls. However, treatment with either TT or AP just effected on Leydig and spermatogonia cell numbers significantly. Hormonal level of testosterone, LH and FSH showed considerable increase in AT group compared to other groups except FSH level in AT group compared to AP group. The weight of prostate and testes were not significantly altered in none of the groups; nevertheless, prostate had a mild increase of weight in all treated groups. Our results for body weight were consistent with the study performed by Sujith et al. on the toxicity of AP in Wistar rats, and inconsistent with Sharma et al. study on the aphrodisiac effects of AP on male rats (28, 29).

A traditional Iranian, northern Khorasan, remedy for enhancing male sexual activity is consumption of a mixture of both TT flower and AP root. TT active components are furostanol saponins called protodioscin and protogracilin, which are responsible for the TT reported biological activities, they up-regulate testosterone and LH and increase libido and spermatogenesis (29-32). It is believed that protodioscin is capable of being converted to dehydroepiandrosterone (DHEA) (13). It is also postulated that TT might increase DHEA levels due to elevating the cAMP level in adrenals (26). The reported main active components of the root of AP are anacyclin, pellitorine, hydrocarolin, inulin and traces of volatile oil and seasamin. To our knowledge, no study has been conducted on its components effects on male fertility (28).

Sperm count, motile sperm count and normal sperm morphology have been reported as indices of male fertility (33, 34). Steroidogenesis and spermatogenesis are two major functions of testis, which are the results of coordination between various cell types, including Sertoli, Leydig and germ cells (35, 36). Hypothalamus-pituitary-gonadal axis increases Leydig cell numbers and stimulates their testosterone production through up-regulating LH; on the other hand, the axis is important for Sertoli cell function and its number (16, 36). Sertoli cells encompass different germ cells which are distributed in seminiferous epithelium, where multiple germ cells are in contact with a single Sertoli cell (36, 37). Testosterone, LH and FSH, are three hormones related to main role in sexual activity. LH stimulates Leydig cells to produce androgen; it also increases Leydig cell number in testis (38). Testosterone regulates the spermatogenesis through phosphorylation of cAMP response element-binding protein (CREB) and its increase has a pivotal role in sperm quantity and quality (8, 39). FSH also triggers the phosphorylation of CREB (40, 41). It has been shown that the presence of mutant CREB in testis of rat causes over 42±5.8% of the seminiferous tubules to have disrupted spermatogenesis, due to apoptosis, causing the loss of 75% of spermatids (10). This proves the important role of phosphorylation of CREB. Testosterone and FSH share similar final activities in different ways, and interestingly testosterone is twofold less efficient in phosphorylating CREB (42). Perhaps, that is why the presence of FSH is necessary for full fertility (43). FSH also causes higher glucose uptake (44, 45) and controls the synthesis of major fuel used by germ cells. FSH prevents apoptosis in spermatogonia and spermatocytes; thus, their viability increases (7, 10). Testosterone induces spermatogenesis and can potentially improve spermatogenesis, if injected exogenously. However, what is really needed for spermatogenesis is intratesticular testosterone; moreover, exogenous testosterone suppresses GnRH, leading to reduced LH, and consequently lower testosterone production by Leydig cells, i.e lower intratesticular testosterone (46, 47).

In our study, the number of Leydig cells increased significantly in all treated groups coordinately with the LH serum level. However, the number of Sertoli cells was elevated significantly only in AT group compared to all other groups, which might be a confirmation for simultaneous increase of serum LH, FSH and testosterone levels compared to other studied groups. Spermatid and spermatogonia numbers increased significantly in all three groups, which might be due to higher testosterone and LH levels; however, their number in AT group was significantly higher than other groups, which can be due to the increased number of Sertoli cells and higher sexual hormonal levels in this group.

In conclusion, TT and AP have positive effects on sexual parameters and sexual hormonal levels in male rats. However, the mixture of AP and TT, as prescribed in traditional medicine of northern Khorasan has considerably remarkable effects on sexual parameters of Wistar rats including enhanced sperm quality, sexual hormonal levels and histoarchitecture of male Wistar rats. The extracts seem to have combined effects. Thus, further studies should be performed on different doses and also to find active agents and their effective combinations. Both TT and AP have been reported to be non-toxic and have no side effects; accordingly, they are safe choices for drug purposes. These findings and future studies based on these results can lead to new drugs for male sexual dysfunction.
